# Early Identification of Hearing Loss and Language Development at 32 Months of Age

**DOI:** 10.3390/ohbm3040008

**Published:** 2022-10-24

**Authors:** Anne B. Harris, Elizabeth Seeliger, Christi Hess, Allison L. Sedey, Kayla Kristensen, Yen Lee, Winnie Chung

**Affiliations:** 1 Waisman Center, University of Wisconsin-Madison, Madison, WI 53705, USA; 2 Wisconsin Department of Health Services, Madison, WI 53703, USA; 3 Speech, Language, and Hearing Sciences, University of Colorado-Boulder, Boulder, CO 80309, USA; 4 Colorado School for the Deaf and the Blind, Colorado Springs, CO 80903, USA; 5 Edgewood College, Madison, WI 53711, USA; 6 Veterans Healthcare System of the Ozark, Fort Smith, AR 72917, USA; 7 National Center on Birth Defects and Developmental Disabilities, Centers for Disease Control and Prevention, Atlanta, GA 30329, USA

**Keywords:** hearing loss, language, expressive vocabulary, early hearing diagnosis

## Abstract

This study examines the relationship between the early identification of hearing loss and language outcomes for deaf/hard of hearing (D/HH) children, with bilateral or unilateral hearing loss and with or without additional disabilities. It was hypothesized that hearing loss identified by 3 months of age would be associated with better language outcomes. Using a prospective, longitudinal design, 86 families completed developmental instruments at two time points: at an average age of 14.8 months and an average age of 32.1 months. Multiple regression examined how hearing loss identified by 3 months of age contributed to later language outcomes while controlling for developmental level at the first time point. Hearing loss identified by 3 months of age was positively associated with better language outcomes for D/HH children at 32 months of age; however, D/HH children still exhibited language delays, compared to normative scores for same-aged hearing peers for reported measures. Language outcomes of children with unilateral hearing loss were not better than those of children with mild-to-moderate bilateral hearing loss. Children with additional disabilities and more severe bilateral hearing loss had lower language scores than those without.

## 1. Introduction

Permanent congenital hearing loss is one of the most frequently occurring chronic conditions in childhood [1]. Before the widespread implementation of universal newborn hearing screening, parents reported a median diagnosis of hearing loss at 22 months with initiation of early intervention services and fitting of hearing aids at a median age of 28 months [2]. In the United States, each state began implementing early hearing detection and intervention (EHDI) programs by the year 2000 [3]. According to the Centers for Disease Control and Prevention (CDC), in 2019, over 98% of newborns received a hearing screening by 1 month of age. For 62% of the infants who did not pass the hearing screening, a diagnosis of presence or absence of a hearing loss was documented. For 79% of these infants, this diagnosis occurred by 3 months of age. For those children with hearing loss who were enrolled in early intervention, 79% received services by 6 months of age [4].

The Joint Committee on Infant Hearing (JCIH) published a 2019 position statement emphasizing early hearing screening (by 1 month of age), evaluation (by 3 months of age), and intervention (by 6 months of age) for all children [5]. Several recent studies have reported that deaf and hard of hearing (D/HH) children whose hearing loss was identified early in infancy had better language outcomes. Wake and colleagues [6] reported improved receptive and expressive language skills. Yoshinaga-Itano and colleagues [7] examined the vocabulary of children when hearing loss was diagnosed by 3 months of age and early intervention begun by 6 months of age. The authors concluded that expressive vocabulary abilities were significantly higher for children who met the JCIH benchmarks for hearing loss identification and intervention, had no additional disabilities, had mild-to-moderate hearing loss, and had mothers with higher levels of education [7].

The purpose of this paper is to examine the relationship between timing of hearing loss identification and language development for children with permanent hearing loss in Wisconsin who were recruited as part of a larger prospective, longitudinal study of developmental outcomes for D/HH children. Identity-first and person-first language are used throughout this paper to reflect terms preferred by both deaf individuals and individuals with hearing loss. To expand on previous research, this study included children with either unilateral or bilateral hearing loss, as well as participants with and without additional disabilities. Other co-variates were examined, such as degree of hearing loss and maternal level of education.

## 2. Materials and Methods

### 2.1. Recruitment and Strategies to Reduce Attrition

In 2008, a partnership between the Wisconsin EHDI program and researchers at the University of Wisconsin-Madison and the University of Colorado-Boulder created the Assessment of Early Intervention Outcomes (AEIOu) study. UW-Madison was the Institutional Review Board (IRB) of record. Recruitment began when children were around 12 months of age. Initially, all D/HH children who were enrolled before 12 months of age in the Wisconsin Part C early intervention program, called the Birth to 3 Program, were eligible to participate in the study. After 2013, inclusion criteria were expanded to include all children reported to the WI EHDI program with a diagnosis of hearing loss regardless of enrollment in the Birth to 3 Program. Exclusion criteria included temporary hearing loss (e.g., related to temporary fluid behind the ears). A study team member contacted eligible families by phone around the time the child was 12 months of age and provided information about the AEIOu study. If the family agreed to participate, they were mailed both the study consent and developmental questionnaires. Because each participant was to be assessed at 2 time points (Phase 1 target age range of 14–20 months, and again at Phase 2 with a target age range of 30–38 months), several strategies were used to reduce attrition and improve timely return of the completed forms. First, 3 weeks after parents were sent the forms, a phone and/or email reminder was sent. If the evaluation forms were not returned, up to 3 contact attempts were made, with the last contact attempt approximately 1 month before the participant was close to aging out of the target age ranges.

### 2.2. Study Protocol

The AEIOu study protocol was designed collaboratively with the CDC-funded National Early Childhood Assessment Project (NECAP) at the University of Colorado-Boulder [7], which assisted in determining the developmental outcome measures used for this study. For Phase 1 of the study, after the caregivers returned the evaluation forms and the signed consent form, a study coordinator called the family to address any questions or concerns related to study participation, witness the caregiver’s consent, and review completed questionnaires to minimize missing data. This process was repeated for Phase 2 of the study. Authorization to release audiologic records was also obtained from families at both time points. Records of diagnostic evaluations were obtained from the diagnostic service agency for all but five participants. Study team audiologists reviewed participant audiological records from Phase 1 and Phase 2, which included electrophysiologic and/or behavioral hearing tests. Families enrolled in therapy services were asked to complete an authorization to release intervention records. Information was obtained on intervention services for most children, including use of hearing aids, however, the effect of type or timing of intervention was not a focus of the analyses reported here.

At both Phase 1 and Phase 2, families were asked to complete demographic and developmental questionnaires. Demographic information included the child’s sex, age, hearing device used, communication method at home, and co-occurring conditions. Maternal demographic information collected included race, ethnicity, presence of parental hearing loss, and parent rating on the effect of co-occurring conditions on the child’s speech and/or language development.

Results from two norm-referenced developmental instruments were analyzed for this paper, the MacArthur-Bates Communicative Development Inventories [8] and the Child Development Inventory [9]. The first evaluation tool measured expressive vocabulary skills. The second evaluation tool queried skills across multiple developmental domains. Both measures have been validated with typically developing children, as well as children with hearing loss [10–12].

Developmental forms were scored at the University of Colorado-Boulder by trained undergraduate students, checked for accuracy by a second scorer, and errors were corrected by consensus.

Quotient scores were calculated using the following equation:

(DevelopmentalAge/ChronologicalAge)×100=DevelopmentalQuotient.

A quotient of 100 indicates a child’s developmental age is exactly commensurate with their chronological age. Quotients below 80 are considered to indicate a clinically significant delay, relative to other children of the same age. Additionally, the manual for the CDI indicates that scores between 70–80 are suggestive of borderline delay [9].

### 2.3. Participants

Data collection occurred over ten years (2009–2019) with a total of 118 children completing Phase 1 and 86 of those children completing Phase 2. This study focuses on the 86 children for whom both Phase 1 and Phase 2 data were available. Although there was some attrition due to losing contact with families or families choosing not to complete Phase 2, children completing Phase 2 did not differ from the group completing Phase 1 on important study group characteristics, such as communication method used at home, hearing status of parents, maternal level of education, types of childhood comorbidities, and degree of hearing loss.

Demographic information for the study cohort of 86 children, as reported at Phase 2, is shown in Table 1, and hearing loss characteristics are reported in Tables 2 and 3. The Wisconsin AEIOu study results represent a subset of the multi-state NECAP data previously reported in 2017 by Yoshinaga-Itano and colleagues [7]. Unlike the Yoshinaga-Itano study, children with either unilateral or bilateral hearing loss were included in the analyses.

Self-identified race in this study cohort was 90% White, and no families reported Hispanic ethnicity. Mothers reported a relatively high level of maternal education (Table 1). The presence of co-occurring conditions in the cohort aligns with previous findings that 30–33% of children with hearing loss are diagnosed with one or more co-occurring conditions [13,14]. In this study, children reported by their parents to have “co-occurring conditions with mild to significant impact on speech/language development” was the group that was considered to have “additional disabilities”, other than hearing loss. All children had hearing loss present at or shortly after birth. The laterality of hearing loss (unilateral or bilateral) is described in Table 2. For the individuals with bilateral hearing loss, hearing levels were further categorized as mild to moderate (pure tone average of 26–55 dB HL in the better ear) or moderately severe to profound (pure tone average of 56 dB HL or greater).

### 2.4. Data Analysis

To examine whether meeting the EHDI recommendation for identification of hearing loss by 3 months of age influenced language development, children were considered to have “met” the 3 months diagnostic recommendation if their hearing loss was identified before 3.5 months of age. Sixty-two children (72%) met this criterion. This categorical variable (“met” or “not met”) was used as one of the predictors for the analyses. Table 3 describes the ages of participants dichotomized into these two categories (“met” and “not met”). Demographic characteristics described in Tables 1 and 2 were not significantly different (*p* > 0.05) for children in these two groups. The only variables that were significantly different between the two groups were age of diagnosis, age of receiving hearing amplification, and age of receiving early intervention, as shown in Table 3.

Outcome variables used in the regression model were the expressive vocabulary quotient from the MacArthur Bates Communicative Development Inventories [8] (Mac_LQ) and the following developmental quotients from the CDI [9]—general development (Gen_Q), social skills (Soc_Q), and language development (Rec_LQ). The MacArthur Bates inventory asks parents to report the number of words or signs the child is using. The CDI focuses on overall development represented by the Gen_Q, which reflects a range of subscales, such as social, motor, and language subscales. Subscales of interest reported here are those related to language development (general usage, reflecting both expressive and receptive language, = Rec_Q) and social skills (Soc_Q), reflecting relational skills. These outcome variables have been previously demonstrated as predictive of later language skills, e.g., [15,16]. All measures were normed on populations, including those with hearing loss and across all age ranges of participants in this study. First, means and SDs were determined for quotients for each of these developmental subscales (shown for both Phase 1 and Phase 2 in Table 4). Children demonstrate variability in language development during the early childhood period, as reflected by the differences in norms for different age ranges of the developmental instruments [8,9]. Target age ranges were selected (Phase 1: 14–20 months, Phase 2: 30–38 months) to measure early language development at two distinct ages, and to support data collection when families completed instruments at different time points.

To examine the impact of co-occurring conditions (“additional disabilities”), 2 groups were created for analysis using parent-reported impact of co-occurring conditions on speech/language development. The first group was composed of children with no additional co-occurring conditions or who have a condition that parents reported as having no impact on speech/language development. The second group was composed of children with additional conditions that parents reported had a mild-to-significant impact on speech/language development.

The transition regression model [17] was used to investigate the relationship between having hearing loss identified by 3 months of age and developmental outcomes at Phase 2 (assessed by caregiver report at an average age of 32 months) while controlling for the developmental level at Phase 1 (assessed by caregiver report at an average age of 15 months). The model included the child’s scores obtained during Phase 1 converted to developmental quotients, considered the “starting point” as a co-variate to account for its influence on later developmental outcomes. Other co-variates included presence of co-occurring conditions reported to impact speech/language development, maternal education (bachelor’s degree or above), and 3 categories of hearing loss (unilateral, bilateral mild to moderate, and bilateral moderately-severe to profound), as defined in Tables 1 and 2. Since our relatively small sample size limited the power for the analysis, we also assessed the effect size to determine the strength of the relationship between the independent and dependent variables [18]. All analyses were performed using R [19].

As a follow up to the previous analyses, we applied hierarchical regression to determine if the age of intervention and the age of amplification could contribute significantly to the variation in developmental outcomes when children’s age of hearing diagnosis and other covariates were considered. Hierarchical regression is commonly used to evaluate the contributions of a variable above and beyond previously entered variables [20]. The order in which variables are examined reflects the fact that any intervention, including aided hearing, would only be initiated after diagnosis.

## 3. Results

The subscale means and SDs are shown in Table 4 as standardized quotients for three developmental subscales of the CDI and also for the MacArthur Communicative Development Inventories. Mean quotients for the entire study cohort were in the normal range (above 80) for all of these developmental outcomes at Phase 1 (when the average age of the children was 15 months). Cohort means were lower at Phase 2 (when the average age of the children was 32 months) than at Phase 1 for all outcomes and fell below 80 for both the CDI language quotient (Rec_LQ) and the MacArthur vocabulary quotient (Mac_LQ), indicating the average outcome for these D/HH children was below the age norm.

As seen in Table 5, the regression models show that hearing loss identification by 3 months of age had small effect sizes (*f*^2^ ≥ 0.02) [21] for associations with better developmental quotients at Phase 2 (group average age of 32 months; Mac_LQ; and Rec_LQ, Soc_LQ, Gen_Q from the CDI). Most of the corresponding *p* values were less than or close to 0.05 after controlling for the other covariates. This model also demonstrates that the developmental quotient at Phase 1 was highly correlated with the same quotient at Phase 2 (*p* < 0.001) for most developmental quotients. After controlling for the developmental score at Phase 1, as well as the other covariates in the analysis model, hearing loss identification by 3 months of age was still significantly related to better outcomes at Phase 2. There were no interactions between identification by the age of 3 months and any of the other covariates, indicating identification of hearing loss by the age of 3 months was independently related to outcomes measured.

The identification of hearing loss must come before intervention for hearing loss can be initiated. Utilizing a hierarchical regression method, neither the age of intervention nor the age of amplification had a significant relationship with developmental quotients at Phase 2 when the identification of hearing loss by age 3 months and covariates were controlled (entered in the model first). The following analyses focused only on the models using hearing loss identified by age 3 months.

Of additional interest is that laterality of hearing loss (i.e., unilateral versus bilateral hearing loss) did not have a separate relationship with these developmental quotients. However, having a moderately severe-to-profound bilateral hearing loss was associated with lower developmental quotients for the four outcomes shown on Table 5. As expected, children with additional disabilities reported to affect speech/language development had lower scores for the MacArthur LQ and the general development quotient on the CDI. This type of regression analysis made it possible to see the separate relationships with each of these predictors when the other covariates were controlled in the model.

**Figure 1.**
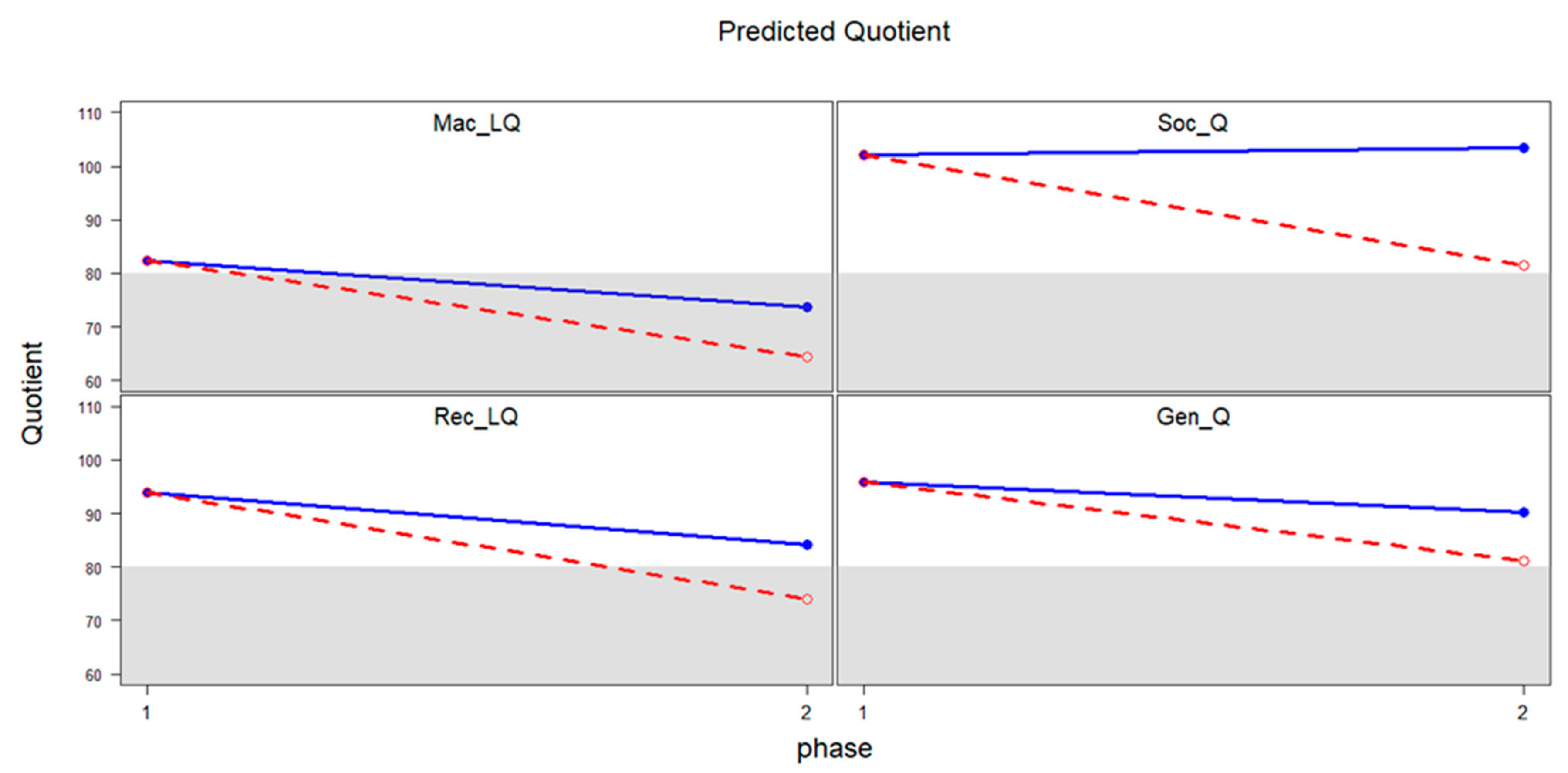
Predicted mean developmental quotients around 32 months of age when hearing loss was identified before and after 3 months of age. The number 1 represents the developmental quotient at Phase 1 when children were around 15 months of age, considered to be their starting point. The number 2 represents the developmental quotient at Phase 2 when children were around 32 months of age. Solid (blue) lines show the predicted mean when hearing loss was identified by three months of age, and the dashed (red) lines depict the predicted mean when hearing loss was identified after three months of age. The shaded area shows the range < 80 for quotients indicative of developmental delay.

Predictive mean values at Phase 2 were computed to visualize the longitudinal trajectory when hearing loss was identified before or after 3 months using the four developmental quotients in Table 5—Soc_Q, Gen_Q, Rec_LQ and Mac_LQ (see Figure 1). Since the children with hearing loss identified by age 3 months and the children identified later were different on some non-demographic covariates, predicted mean values were computed either assuming all children were identified by age 3 months or not, to ensure that the effect of covariates was considered. The predicted values suggest that children whose hearing loss was identified by 3 months of age had a smaller decrease in their overall score between Phase 1 and Phase 2 for these four outcome variables.

## 4. Discussion

Analyses reported here consistently demonstrate that hearing loss identified by 3 months of age is associated with better developmental outcomes, at least up to an average of 32 months of age, as measured by the MacArthur vocabulary quotient (Mac_LQ) and three subscales of the CDI (Gen_Q, Rec_LQ and Soc_Q) shown in Table 5. First and foremost, as promoted in the JCIH 2019 position statement and other studies showing that early hearing loss identification is a protective factor for child development, these results reinforce the role of the timely identification of hearing loss in supporting language development for children who are D/HH [5]. Unique features of this study include (1) longitudinal data from two time points, where earlier developmental outcomes were controlled for in the analyses, (2) better outcomes that are evident across subscales of two different instruments, and (3) developmental scores for children with unilateral hearing loss were not better than those of children with mild-to-moderate bilateral hearing loss.

Children with additional disabilities and also those with moderately severe-to-profound bilateral hearing loss, predictably had more significant developmental delays than those who had lesser degrees of hearing loss independent of age of identification of hearing loss (Table 5). There were no significant interactions detected between the variables. Our result indicates that early hearing loss identification was associated with improved language outcomes when the other covariates are controlled. By controlling for the developmental level at Phase 1 in the analyses, each participant essentially served as their own control. Although the rate of language development does vary across these age ranges, standardized scores for instruments were age normed by month, so variability in age differences between Phase 1 and Phase 2 were accounted for statistically.

Almost all children were enrolled in intervention at some point in the study, since this was initially one of the criteria for study participation. The hierarchical regression indicated that the general age of enrollment in intervention services (i.e., age reported by parents that child started intervention services and not stratified by the type of intervention provided) did not have a significant unique contribution to the longitudinal language outcomes in this cohort when the age of early hearing loss identification was controlled. This finding may relate to the heterogeneity of interventions that participants received, which were not exclusive to language. For example, fewer than half of the children in this cohort received speech-language pathology services at either Phase 1 (45%) or Phase 2 (49%). The timing of audiological and speech and language intervention [22,23], fitting of amplification [24], and timing of enrollment with providers with specialized training and experience with children with hearing loss and in home-based auditory/oral programs, home-based total communication programs, and home-based specialty programs for children with hearing loss [25–27] have been demonstrated in recent research to impact communication outcomes. However, it is important to note that timing of hearing loss diagnosis and intervention are temporally related, and data in Table 3 showed that children who were diagnosed earlier also tended to receive intervention earlier. As demonstrated in the hierarchical regression, diagnosis by age 3 months emerged as the first-occurring independent association with better language outcomes for this cohort.

In general, maternal educational level in this Wisconsin cohort was high. Sixty-four percent of mothers reported having a bachelor’s degree or higher education. Maternal educational level had significant (*p* < 0.05) associations with the CDI language quotient (Rec_LQ), CDI social skills quotient (Soc_Q), and CDI general development quotient (Gen_Q) but not for the MacArthur vocabulary quotient (Mac_LQ) (shown in Table 5). Family socio-economic status (SES) can be estimated from parent education level or occupational skill level. These factors have been found to predict language development in children with normal hearing [28,29], as well as children with hearing loss [30]. More specifically, an early study by Dollaghan and colleagues [31] and a later study by Cupples and colleagues [32] found that parental education level statistically predicted receptive language skills in a cohort of typically developing, normal hearing 3-year-old children. When maternal educational level was examined in a cohort of 5-year-old children with hearing loss by Cupples and colleagues [33], the authors continued to find maternal education was a significant predictor of language outcomes. The current study’s findings are consistent with this previous research.

Using statistical modeling, predictive values in Figure 1 show that language quotients decreased on the standardized assessments between Phase 1 (average age of 15 months) and Phase 2 (average age of 32 months), both for children who were identified with hearing loss by 3 months of age and for children identified later. Despite better language outcomes associated with early hearing loss identification and access to intervention, D/HH children still lagged behind their hearing peers. Yoshinaga-Itano and colleagues [7] noted the vocabulary quotient of D/HH children who were identified and received intervention early was less than the expected mean for the participants’ age. Tomblin and colleagues [34] found early access to hearing amplification improved the language outcomes of D/HH children. However, on average, D/HH children still showed lower language levels, when compared to normal hearing peers matched on age and socioeconomic status. Table 4 shows that, when compared to typically developing peers, D/HH children’s developmental quotients at Phase 1 were lower than the expected mean of 100 but the mean was still in the range considered “average” (above 80). This finding is consistent with previous research [7,34]. The depressed scores at Phase 2 may reflect the increased communication demands as children grow older. The lower scores suggest the importance of identifying additional factors that may contribute to developmental delays in D/HH children and understanding the impact of other factors, such as etiology of hearing loss, mode of communication, use of amplification, and the amount and type of intervention related to hearing loss. Parent report measures have been shown to be valid and reliable methods of evaluation of language abilities in children with language delays [35]. However, future research will benefit from a combination of parent report and direct assessment measures to continue in-depth examinations of language profiles for D/HH children across domains of communication development and as children progress through school.

### Limitations and Strengths

While the small cohort of children in this study appears to be a limitation, the longitudinal nature of the study provides an advantage allowing for extensive data collection over a longer time period for the same children, yielding analyses with multiple outcome variables, controlling for a variety of potential covariates. Though 118 children were initially enrolled, only 86 children completed both phases of the data collection during the study period; however, there were no significant differences in the demographic characteristics of those who completed both phases versus those initially enrolled. This suggests that attrition did not affect the cohort characteristics. Due to the uniform methods of recruitment and data collection, which initially included only families enrolled in the Birth to 3 Program, this study cohort cannot be considered representative of the population of children and families in Wisconsin. In addition, the maternal educational level of this cohort is relatively high in that 64% of the mothers had a bachelor’s or higher degree, which may be considered a proxy for a higher than average socio-economic status. These results may not be generalizable to D/HH children whose mothers have less education. At the same time, having a study cohort whose mothers generally have a higher education exposes the fact that D/HH children still lag behind in several developmental areas, even with a more highly educated group of parents. This suggests that while maternal education may predict improved developmental outcomes for D/HH children, these children are still at developmental risk. Although the current study collected some information on intervention, this variable was not significantly independently related to better language outcomes in this analysis. It is important for future studies to examine the impact of timing, amount, and type of intervention, in addition to early identification of hearing loss.

This study also included children with unilateral hearing loss and additional disabilities, which is a departure from other similar studies. In this study, the developmental trajectory of a child with unilateral hearing loss was similar to that of children with mild-to-moderate bilateral hearing loss. As a group, early diagnosis predicted better language outcomes for children assessed in this inclusive study sample.

## 5. Conclusions

When hearing loss was identified by the recommended 3 months of age, children who are D/HH scored higher on standardized measures, compared to children whose hearing loss was identified later across several domains of parent-completed developmental instruments at an average age of 32 months. Despite better language and developmental outcomes associated with early hearing loss identification and access to intervention for most participants, standardized developmental scores for this cohort of D/HH children still lagged behind their hearing peers. Additional analyses using fitted values derived from the regression results suggest that if hearing loss was identified by 3 months of age, language skills did not fall as far behind, compared to participants whose hearing loss was identified later.

In this cohort, measured outcomes for children with unilateral hearing loss were not better than those children with mild bilateral hearing loss. Children with additional disabilities and moderately severe-to-profound bilateral hearing loss experienced more significant delays. The association between early hearing loss identification and better language outcomes was significant within a cohort of children with known existing protective factors (e.g., access to early intervention, mothers with high levels of education). Further research is needed with culturally and linguistically diverse groups to examine associations between early identification of hearing loss and language outcomes.

## Figures and Tables

**Table 1.** Participant demographic characteristics.

Demographic Characteristic	Ages: Mean (SD) and Range, or Number of Participants (%) *n* = 86

**Age at Phase 1:**	
Mean (SD)	14.8 months (2.2 months)
Range	10–26 months

**Age at Phase 2:**	
Mean (SD)	32.1 months (2.8 months)
Range	29–45 months

**Sex**	
Male	39 (45%)
Female	47 (55%)

**Race**	
White	77 (90%)
Asian	1 (1%)
Black/African American	2 (2%)
Other	6 (7%)

**Maternal education level**	
Less than high school	1 (1%)
High school graduate or equivalent degree	18 (21%)
Vocational	7 (8%)
Associate	5 (6%)
Bachelor’s degree (BA or BS)	37 (43%)
Graduate school	18 (21%)

**Co-occurring conditions’ impact on speech language development reported by parents**	
No impact	56 (65%)
Mild to significant impact	30 (35%)

**Table 2.** Characteristics of hearing loss and age of hearing loss identification, amplification, intervention, and communication mode used.

Hearing Characteristics	Number of Participants (%) *n* = 86

**Laterality and degree of hearing loss**	
Unilateral	20 (23%)
Bilateral	66 (77%)
Degree of hearing loss in better ear *(the following distribution is for bilateral only)*	
Mild to moderate (26–55 dB HL)	29 (44%)
Moderately severe to profound (>55 dB HL)	32 (48%)
Missing data/Not available	5 (8%)

**Type of amplification**	
None	13 (15%)
Hearing aids	47 (55%)
Cochlear implant	17 (20%)
Cochlear implant and hearing aid	2 (2%)
Bone conduction hearing aid	6 (7%)
Missing data/Not available	1 (1%)

**Presence of auditory neuropathy**	
No	82 (95%)
Yes	4 (5%)

**Child’s mode of communication**	
Spoken language only	26 (30%)
Spoken language with occasional sign language	28 (33%)
Both sign language and spoken language	16 (19%)
Sign language only	5 (6%)
Cued speech	1 (1%)
None yet	8 (9%)
Missing data/Not available	2 (2%)

**Hearing status of parents**	
Normal hearing in both parents	79 (92%)
One or both parents are deaf or hard of hearing	7 (8%)

**Table 3.** Age and hearing characteristics for participants with hearing loss identified by 3 months and participants with hearing loss identified after 3 months.

Hearing Characteristic	Identified by 3 Months (*n* = 62)	Identified after 3 Months (*n* = 24)

**Age at Phase 1**		
Mean (SD)	14.5 months (1.9 months)	15.5 months (2.8 months)
Range	10–19 months	12–26 months

**Age at Phase 2**		
Mean (SD)	32.0 months (2.7 months)	32.3 months (3.3 months)
Range	29–45 months	29–41 months

**Age hearing loss identified**		
Mean (SD)	1.8 months (0.7 months) *	6.5 months (2.8 months) *
Range	0.5–3 months	3.5–15 months

**Age hearing loss aided**		
Mean (SD)	4.2 months (2.3 months) *	9.6 months (3.4 months) *
Range	1–13 months	5–18 months

**Age early intervention started**		
Mean (SD)	4.1 months (2.9 months) *	7.8 months (3.4 months) *
Range	0.5–13 months	2–17 months

The independent *t*-test was used to determine whether there was a difference between participants identified by and after 3 months on these variables

* *p* < 0.001.

**Table 4.** Mean and SD of the developmental quotients at each test phase (*n* = 86).

	Phase 1		Phase 2	
	Mean	SD	Mean	SD
Rec_LQ	93.13	21.18	77.34	26.84
Soc_Q	100.91	22.60	92.54	38.66
Gen_Q	94.72	16.20	83.27	23.36
Mac_LQ	81.06	22.19	71.68	19.26

Rec_LQ: CDI language. Soc_Q: CDI social skill. Gen_Q: CDI general development. Mac_LQ: MacArthur vocabulary.

**Table 5.** Transition regression analysis result of multiple factors on selected developmental quotients.

	Mac_LQ			Rec_LQ			Soc_Q			Gen_Q		

	B	SE	*p*	B	SE	*p*	B	SE	*p*	B	SE	*p*

(Intercept)	38.70	(7.53)	<0.001	19.27	(12.37)	0.124	21.14	(21.19)	0.322	31.06	(13.56)	0.025
ID_by_3	**9.28**	**(3.62)**	**0.013**	9.98	(5.29)	0.064	**21.92**	**(9.12)**	**0.019**	**9.02**	**(4.41)**	**0.045**

Bilateral hearing loss (Yes)	0.15	(3.88)	0.969	0.00	(5.80)	1.000	3.69	(10.00)	0.714	5.40	(4.76)	0.262

Severity of bilateral hearing loss	−6.78	(3.58)	0.062	**−11.35**	**(5.25)**	**0.035**	−17.47	(9.06)	0.058	**−10.32**	**(4.26)**	**0.019**

Maternal education (Bachelor’s degree or above)	4.14	(3.29)	0.212	**13.70**	**(4.73)**	**0.005**	**18.03**	**(8.21)**	**0.032**	**9.81**	**(3.92)**	**0.015**

Presence of co-occurring conditions (Yes)	**−15.85**	**(3.63)**	**<0.001**	−10.60	(5.68)	0.067	−12.75	(9.75)	0.196	**−11.73**	**(4.79)**	**0.017**

Score at 15 months	**0.36**	**(0.08)**	**<0.001**	**0.56**	**(0.11)**	**<0.001**	**0.54**	**(0.18)**	**0.004**	**0.48**	**(0.13)**	**<0.001**

Effect size of ID_by_3 (*f*^2^)			0.040			0.026			0.059			0.033

Note: Score at 15 months = developmental outcome scores of each measurement at an average age of 15 months (Phase 1); B = estimated regression coefficient, SE = standard error; items significant at *p* < 0.05 are bolded.

## Data Availability

The data are not publicly available due to privacy restrictions. Some de-identified data are available on request due to privacy restrictions.
